# Review of the effectiveness of neuromuscular electrical stimulation in the treatment of dysphagia – an update

**DOI:** 10.3205/000310

**Published:** 2022-06-14

**Authors:** Simone Miller, Katharina Peters, Martin Ptok

**Affiliations:** 1 Klinik für Phoniatrie und Pädaudiologie, Hannover, Germany

**Keywords:** swallowing, dysphagia, neuromuscular electrical stimulation (NMES), deglutition

## Abstract

**Background:**

Neuromuscular electrical stimulation (NMES) has been used as a treatment option in the therapy of dysphagia for several years. In a previous review of the literature, it was concluded that NMES might be a valuable adjunct in patients with dysphagia and in patients with vocal fold paresis. However, due to different stimulation protocols, electrode positioning and various underlying pathological conditions, it was difficult to compare the studies which were identified and it was concluded that more empirical data is needed to fully understand the benefits provided by NMES. The purpose of this systematic review is, therefore, to evaluate recent studies regarding a potential effectiveness of transcutaneous NMES applied to the anterior neck as a treatment for dysphagia considering these different aspects.

**Method:**

For this systematic review, a selective literature research in PubMed has been carried out on 5^th^ May 2021 using the terms *electrical stimulation AND dysphagia* and screened for inclusion criteria by two reviewers in Rayyan. The search resulted in 62 hits.

**Results:**

Studies were excluded due to their publication language; because they did not meet inclusion criteria; because the topical focus was a different one; or because they did not qualify as level 2 studies. Eighteen studies were identified with varying patient groups, stimulation protocols, electrode placement and therapy settings. However, 16 studies have reported of beneficial outcomes in relation with NMES.

**Discussion:**

The purpose of this systematic review was to evaluate the most recent studies regarding a potential effectiveness of NMES as a treatment for oropharyngeal dysphagia considering different aspects. It could generally be concluded that there is a considerable amount of level 2 studies which suggest that NMES is an effective treatment option, especially when combined with TDT for patients with dysphagia after stroke and patients with Parkinson’s disease, or with different kinds of brain injuries. Further research is still necessary in order to clarify which stimulation protocols, parameters and therapy settings are most beneficial for certain patient groups and degrees of impairment.

## Zusammenfassung

**Hintergrund:**

Bereits seit einigen Jahren wird die neuromuskuläre Elektrostimulation (NMES) auch zur Behandlung von Schluckstörungen eingesetzt. Ein vorausgegangener Übersichtsartikel zu ihrer Wirksamkeit legte bereits den Schluss nahe, dass die NMES einen gewinnbringenden Zusatz in der Therapie der Dysphagie und bei Stimmlippenparesen darstellen kann. Ein Vergleich der damals vorliegenden Studien war allerdings aufgrund von abweichenden Stimulationsprotokollen, der Positionierung der Elektrodenanlagen und verschiedentlicher Grunderkrankungen nur eingeschränkt möglich. Die Arbeitsgruppe schlussfolgerte, dass weitere empirische Daten notwendig sind, um die Vorteile der NMES-Behandlung abschließend bewerten zu können. Ziel dieser systematischen Übersichtsarbeit ist es daher, neuere Studien über potentielle Effekte der transkutanen NMES (der vorderen Halsmuskeln) für die Dysphagietherapie zu bewerten.

**Methode:**

Am 5. Mai 2021 wurde eine Literaturrecherche in Pubmed mit den Suchkriterien *electrical stimulation AND dysphagia* durchgeführt und anschließend in Rayyan von zwei Bewertern auf ihre Einschlusskriterien überprüft. Die Suche ergab 62 Treffer.

**Ergebnisse:**

Aufgrund einer abweichenden Publikationssprache, einer differierenden Applikationsart, einer unterschiedlichen inhaltlichen Ausrichtung oder einem niedrigeren Evidenzlevel als 2 wurden 44 Studien ausgeschlossen. Es verblieben 18 Studien, die teilweise unterschiedliche Patientengruppen, Stimulationsprotokolle, Elektrodenanlagen und Therapiepläne untersuchten und dennoch in 16 Fällen von nutzbringenden Outcomes in Verbindung mit der NMES-Therapie berichteten.

**Diskussion:**

Das Ziel dieser Übersichtsarbeit war es, eine Bewertung der aktuellen Studienlage hinsichtlich des Nutzens der NMES in der Therapie von oropharyngealen Dysphagien vorzunehmen. Die durchgeführte Literaturrecherche legte eine beachtliche Anzahl an Level 2 Studien offen, die der NMES einen Gewinn in der Behandlung von Dysphagiepatienten zusprechen. Dies gilt insbesondere für Patienten nach Schlaganfall, mit M. Parkinson oder mit verschiedenen Schädel-Hirn-Verletzungen und in Kombination mit einer traditionellen Dysphagietherapie (TDT). Es fehlen jedoch nach wie vor empirische Daten zur Effektivität verschiedener Stimulationsprotokolle, Stromparameter und Therapiesettings, insbesondere im Hinblick auf unterschiedliche Patientengruppen und Schweregrade.

## Background

The term ‘dysphagia’ refers to swallowing disorders, which can be caused by a variety of underlying conditions. Common disorders associated with dysphagia are of neurological (e.g. stroke or Parkinson’s disease) or structural (e.g. head and neck cancer) origin. Dysphagia is associated with symptoms like drooling or leaking from the oral cavity during food intake, coughing before, during or after swallowing, but also the so-called “silent aspiration” of food, liquids or saliva into the airways. Dysphagia often results in dehydration, malnutrition, airway obstructions, pneumonia and an increasing risk of mortality associated with aspiration pneumonia [1].

Conservative treatment options, like the traditional dysphagia therapy (TDT) [2] are based on three therapy principles: ‘restitution’ – aiming to restore lost muscle functions; ‘compensation’ – using compensatory strategies, like postural changes to replace lost functions; as well as ‘adaptation’ – using dietary modifications or certain tools in order to enable safe swallowing.

Neuromuscular electrical stimulation (NMES) aims to restore and enhance the motor function of weak muscles as well as enable muscle contraction in order to prevent muscle atrophy. In recent years, numerous studies have investigated NMES as a treatment option for oropharyngeal dysphagia, but study protocols, patient groups, electrode placements and treatment protocols differ greatly.

In a previous review of the literature by Miller et al. [3], it was concluded that there is evidence that NMES is a valuable adjunct in patients with dysphagia and in patients with vocal fold paresis. However, due to different stimulation protocols, electrode positioning and various underlying pathological conditions, it was difficult to compare the studies which were identified, and it was concluded that more empirical data is needed to fully understand the benefits provided by NMES.

The purpose of this systematic review is, therefore, to evaluate the latest studies regarding a potential effectiveness of transcutaneous NMES applied to the anterior neck as a treatment for dysphagia considering these different aspects.

## Methods

A selective literature research in PubMed (https://pubmed.gov) has been carried out on 5^th^ May 2021, using the terms: *electrical stimulation AND dysphagia*. A filter was applied determining a time frame from 2014/1/1 until 2021/5/6 (the cut-off date corresponds with our previous review [3]). More filters were used to only include clinical trials and randomized controlled trials. The search resulted in 62 hits.

Those 62 results were transferred to Rayyan (https://www.rayyan.ai/) for systematic and independent screening by two reviewers as to whether an article qualified to be “included”, “excluded” or “undecided”. Only studies were included which applied transcutaneous electrical stimulation and investigated effects related to dysphagia, published in either English or German. The software then created a count for each category as well as the conflicts between reviewers for further classification.

After determining the level of evidence (Oxford Centre of Evidence-Based Medicine) for each study, only those studies were selected which qualified as at least level 2 studies. After the initial screening of the abstracts by the two reviewers, existing “conflicts” were resolved by consulting the full texts of the articles to check if inclusion criteria were truly met and agreed on by both reviewers. After this, all full-text articles (of studies marked as “included”) were analysed.

Seven studies were excluded due to their publication language and 33 because they did not meet inclusion criteria, e.g. NMES was not applied transcutaneously or because the topical focus was a different one. Four further studies were excluded because they were neither randomized trials nor observational studies with dramatic effects. Eighteen studies remained.

### Statistical analysis

A statistical analysis (e.g. forest plot) could not be carried out due to the inhomogeneity of study protocols (see Results) and inappropriate endpoint definition.

## Results

From the selected articles, the following information was extracted and compared (Attachment 1):

- Authors, year, title
- Participants and comparisons
- NMES device and application period
- Electrode placement and NMES parameter
- Action during stimulation
- Swallowing outcome measure
- Results
- Level of evidence

### Participants and comparisons

Most of the studies investigated patients with dysphagia after subacute stroke (6 of 18 studies) [4], [5], [6], [7], [8], [9] acute stroke (5 of 18 studies) [10], [11], [12], [13], [14] or acute and sub-acute stroke (1 of 18 studies) [15]. One study investigated the effects of NMES on dysphagia due to head and neck cancer (1 of 18 studies) [16], one paper reported on patients with Parkinson’s disease (1 of 18 studies) [17], three more studies investigated different kinds of diseases (e.g. different kinds of brain injuries) leading to dysphagia [18], [19], [20]. One study investigated healthy older adults [21].

9 studies controlled the investigations using a control group treated by TDT and/or swallowing maneuvers alone and no sham NMES [4], [7], [8], [9], [10], [11], [12], [14], [15] whereas 6 studies controlled the investigations using a control group treated by TDT and/or swallowing maneuvers with sham stimulation [5], [13], [16], [17], [18], [19]. Three further studies compared different treatment options: the study of Oh et al. [6] compared the effects of different electrode placements, the study of Poorjavad et al. [21] compared the effects of NMES with the effects of head lift exercises, and the study of Ortega et al. [20] compared the effects of NMES with effects of a sensory stimulation combined with capsaicin.

### Application period

Patients were generally treated once per day (range: 1–3 times a day) and five days a week (range: 3–7 days a week). A therapy session was reported to last for 15 to 60 minutes over a period of two, three, four, six, eight or twelve weeks.

### Electrode placement

Electrodes are most commonly placed to either target the mylohyoid and geniohyoid muscles, which are located between the mandible and the hyoid bone and are generally referred to as ‘suprahyoid muscles’, or the thyrohyoid, omohyoid, sternohyoid and sternothyroid muscles, which are located below the hyoid bone and are referred to as ‘infrahyoid muscles’.

Five of the 18 selected studies investigated stimulation in the suprahyoid region [8], [9], [10], [16], [21] and five studies investigated effects of stimulation of the infrahyoid region [4], [5], [14], [17], [20]. Most studies used a horizontal electrode arrangement, whereas Huang et al. tried a vertical electrode arrangement [14]. Four studies investigated the effect of a stimulation protocol applying NMES to both the suprahyoid and the infrahyoid muscle groups together: two studies used two pairs of electrodes each placed horizontally [15], [19] and two studies placed two pairs of electrodes vertically along the midline [7], [11]. One study compared the effects of suprahyoid vs. infrahyoid electrode placement during NMES on dysphagia [6]. Three studies used different electrode locations [12], [13], [18].

### NMES parameter

The frequency at which NMES was applied ranged from 25 to 120 Hz, with 80 Hz being used most often. All studies used a low-frequency current, which is primarily known to stimulate the nerves in order to facilitate muscle contractions [22]. The pulse width of the current was mostly set to 300 ms or 700 ms, but ranged from 300 ms to 1000 ms. Stimulation intensity varied greatly across all studies. Eleven studies [5], [6], [7], [9], [10], [14], [15], [16], [17], [19], [21] have reported using stimulation intensities eliciting muscle contractions (above motor threshold), whereas the individual protocols of the stimulation above motor threshold ranged from a “perceived muscle contraction” [9] or “comfortable contraction” [15], [16] to “strong contraction” [5], [17] or even “maximum tolerable contraction” [14], [19], [21].

Four studies stimulated at sensory threshold [12], [13], [18], [20], an intensity level at which a “sensory sensation” is reached which is insufficient to produce muscle contractions. If specified, sham stimulation was applied at 1 mA [5] or 0.1 mA [18].

### Action during stimulation

Eight studies applied NMES at rest, without any simultaneous voluntary muscle action [10], [11], [12], [13], [14], [18], [20], [21]. In the remaining studies, NMES was applied during TDT [4], [7], [19], while patients performed swallows [9], [15], swallowing maneuvers (especially effortful swallows) [5], [6], [16], [17] or swallowing strengthening exercises [8].

### Swallowing outcome measures

Fourteen studies relied on *instrumental examination devices* to quantify the functional recovery of swallowing [4], [5], [6], [7], [8], [9], [10], [13], [14], [15], [16], [17], [19], [20], like videofluoroscopic swallowing studies (VFSS) or outcome of fiberoptic endoscopic evaluation of swallowing (FEES) [4], [13]. Several scales were used in order to assess and quantify swallowing abilities (Table 1 (Tab. 1)): 

Some studies relied on clinical assessments or screenings like

- Water Swallow Test (WST) [15]
- Kubota Water Drinking Test [11]
- Repetitive Saliva Swallowing Test (RSST) [15]
- Volume Viscosity Swallow Test (VVST) [9] or
- Mann Assessment of Swallowing Ability (MASA) [13]

Questionnaires evaluating the swallowing-related quality of life (HNCI – Head and Neck Cancer Inventory) [16], the level of patient satisfaction in relation to treatment (Likert scale) [19], the self-perception of dysphagia (Eating Assessment Tool, EAT-10) [20] or the psychological state (Hamilton Anxiety scale and Hamilton Depression Scale) [11] have been used in some studies. Clinical outcomes determining the type of diet, oral and nutritional intake, body weight, the need for postural compensations and the duration of the dysphagia training have also been reported by some studies. Other specific outcome parameters represent

- the cough latency times against a 1% citric acid mist [18]
- pre- and post-therapy surface electromyography (sEMG) during water swallowing [21]
- pressure parameters of esophageal manometry [19]

## Discussion

As the results show, even though many studies exist which investigate the effects of NMES on dysphagia, study protocols and procedures are very inhomogenous and a comparison is very difficult.

Overall, of the 14 studies which based their results on instrumental and objective examination methods, twelve studies reported (limited) positive outcomes for dysphagia when treated with NMES in combination with TDT and/or effortful swallowing [4], [5], [6], [7], [8], [10], [13], [14], [15], [17], [19], [20]. Ten of these twelve studies used a comparison group treated by TDT (with or without sham NMES) [4], [5], [7], [8], [10], [13], [14], [15], [17], [19] and, therefore, indicate benefits of the combined dysphagia therapy with NMES. Benefits of the combined treatment were associated with i.e.: an improvement of the oral and the pharyngeal transit time (VDS) [5], [10], an increased hyoid bone movement (VFSS: Image J program) [5], [17], a reduction of aspiration (PAS or DOSS) [4], [5], [15], [17], [19] and an improvement in oral feeding (VDS: FOIS scale) [4], [19]. The remaining four studies based their findings on clinical assessments or screenings. Three of these reported more positive outcomes for dysphagia or dysphagia-related parameters [11], [12], [18] when treated with NMES. One study did not find any positive change in the activity of suprahyoid muscles during swallowing after an intervention with NMES [21]. It has to be taken into account that this study examined healthy older adults which may not benefit from the treatment in the same way due to a lack of swallowing impairments. One further study reported negative effects in relation with NMES treatment on patients with head and neck cancer [16]. As swallowing exercises alone were not able to offer great benefit to this patient group either, the authors discuss whether current behavioral therapies are generally limited in reversing long-term (chronic) dysphagia in these cases of moderate to severe dysphagia caused by radiation therapy [16]. It might also be challenging that head and neck cancer patients, especially in a large study like this, are very homogenous with regard to tumor size, missing structures, lymphatic gland removal, treatment type etc. Furthermore, other study groups have suggested that due to the more mechanical (muscular and structural) cause of dysphagia in patients with head and neck cancer, therapy generally focusses on compensating for muscle loss or scared tissue by increasing the sensitive afferent information and/or activating the remaining muscle groups to produce swallowing movements instead. The way NMES is applied during most of the study set-ups, it targets primary muscle-nerve units and may therefore be more effective in neurogenic dysphagia than head and neck cancer patients [23]. In addition, as this review shows, more studies have investigated the effects of NMES on neurogenic dysphagia, whereas not as many studies have investigated head and neck cancer patients yet.

Taking the studies mentioned above into account, it can generally be concluded that NMES seems to be an effective treatment option when combined with TDT for patients with dysphagia after acute and/or subacute stroke. All twelve studies which investigated this group of patients reported post-treatment benefits in relation with NMES. It still remains unclear, however, how long the reported treatment benefit lasts, i.e. if the effects are short lived or do potentially provide long-term effects. Only few studies have investigated a long-term effect. Guillen-Sola et al. [9] and Terré et al. [19] carried out a three-month follow-up; both studies found positive effects after treatment, with a similar outcome for the experimental group compared to the control group at three-month follow-up. These results indicate that an NMES treatment for the investigated parameters may rather cause immediate or short-term benefits, or, as Terré et al. discussed, may shorten the recovery period [19].

Although there are only a few studies which examined patients with dysphagia following different neurological diseases (e.g. brain injury or Parkinson’s Disease), there is indication in the literature that NMES might be an effective treatment option for these patients, too [17], [18], [19]. In contrast, patients with dysphagia following head and neck cancer were not found to benefit from NMES in the same way, as reported by Langmore et al. [16]. It should be noted, however, that this is the only study in this review investigating this patient group.

As can be seen in the result section, many different stimulation protocols, electrode placements and application periods have been used, which makes it difficult if not impossible to compare these studies directly. Even though Simonelli et al. [4] hypothesized that the adequate duration of stimulation represents a key factor in the effectiveness of NMES therapy, there is not a single study which better defines or investigates this treatment modality.

With regard to electrode placement, stimulation protocols most commonly target the suprahyoid muscles, as they pull up the hyoid and towards the mandible, whereby the larynx is elevated, allowing the epiglottis to close off the larynx and reduce the risk of penetration and aspiration [24]. Four of the seven studies using stimulation protocols targeting suprahyoid muscles reported positive outcomes [6], [8], [10], [15]. The remaining three studies reported negative outcomes [16], [21], or invariable effects [9]. As stated above, it should be taken into account that these remaining studies examined patients with dysphagia following head and neck chancer [16], healthy older adults [21] or only evaluated long-term effects of NMES [9].

Another approach represents the isolated stimulation of the infrahyoid muscles usually combined with anti-resistance swallowing exercises. All six studies using stimulation protocols targeting infrahyoid muscles stated positive or limited positive outcomes [4], [5], [6], [14], [17], [20]. Reviewing the literature, a positive impact of NMES targeting infrahyoid muscles on hyoid bone movement (horizontal and vertical) and on aspiration as quantified by PAS has been shown [4], [5], [17]. Nevertheless, there is an ongoing debate about whether this stimulated movement of the hyoid bone against the normal swallowing mechanism can pose an increased risk of aspiration, or if creating a resistance to intended muscle activity improves muscle strength more efficiently than assistive electrical stimulation [5]. In order to avoid an increased aspiration risk, the isolated stimulation of infrahyoid muscles as well as the simultaneous stimulation of suprahyoid and infrahyoid muscles is usually applied in combination with swallowing exercises only. Accordingly, three of the studies underline the importance of swallowing actions during stimulation of the infrahyoid muscles [4], [5], [17]. This may be a reason why in these studies, patients with significant cognitive deficits and/or restricted ability to swallow voluntarily were excluded [4], [5], [17].

Some studies suggest, however, that an electrode placement in the infrahyoid area might also target the inner laryngeal muscles for vocal fold closure [25], or the thyrohyoid muscle which is also involved in larynx elevation [15], [26]. In these cases, infrahyoidal stimulation is generally used in combination with suprahyoidal stimulation.

Four studies stimulated the suprahyoid and infrahyoid regions simultaneously [7], [11], [15], [19] and also stated that due to the bigger muscle size, the infrahyoid muscles are said to be stronger than the suprahyoid muscles, resulting in a downward movement of the hyoid bone, acting as resistance during swallowing exercises. Three of the four studies using stimulation protocols targeting supra- and infrahyoid muscles reported positive outcomes for NMES alone or in combination with TDT [11], [15], [19], and one study reported about restricted positive effects [7].

The studies which compared the effect of NMES applied to suprahyoid muscles alone or both muscle groups combined (suprahyoid muscles and infrahyoid muscles) with the effect of NMES applied to infrahyoid muscles alone [6], [15] found that these two types of NMES electrode placement have similar effects on improving swallowing functions in general. NMES applied to the suprahyoid region was found to cause a stronger reduction in PAS scores compared to NMES applied to the infrahyoid region [6] and may bring along the additional benefit of improving the moving distance of the hyoid bone anteriorly [15].

Half of the studies investigated here applied NMES during some kind of swallowing action or TDT [4], [5], [6], [7], [8], [9], [15], [16], [17], [19]. It is argued that NMES paired with swallowing exercises is in keeping with the neuroplasticity principle of specificity of training, e.g. a combination may have a long-term effect in reorganization of the human cortex, resulting in the enhancement of brain plasticity/recovery in swallowing control [16]. During voluntary muscle activation, type I fibres usually become active before type II fibres. Isolated muscle stimulation is under debate, as it reverses this order by initially recruiting type II muscle fibres, even though type II fibres are said to produce more muscle force [4]. The application of NMES during swallowing exercises, therefore, aims to recruit both type I and II muscle fibres simultaneously, and with this, in theory, generates a larger swallowing muscle force and enhances the therapeutic effect in comparison to traditional dysphagia treatment (TDT) or NMES exercises alone [4]. This review of the literature, however, shows that six out of eight studies in which no action was performed during stimulation also reported significant improvements in swallowing after treatment [10], [11], [12], [13], [18], [27].

Neuromuscular electrical stimulation (NMES) generally aims to restore and enhance motor function of weak muscles as well as enable muscle contraction in order to prevent muscle atrophy. Stimulation protocols can either be applied at a sensory level or at a motor level.

The sensory approach is said to increase the local sensory input to the central nervous system via the central pattern generators (CPG). It is said to induce the action of swallowing, and therewith elicit both sensory and motor effects [12]. The motor approach, on the other hand, elicits muscle contractions in the targeted muscles, which is seen as a muscle training to improve and enhance muscle strength and prevent atrophy [12].

While Zhang et al. found the sensory approach combined with traditional swallowing therapy to be more beneficial than the motor approach, other study groups like Park et al. [5] concluded that the motor approach in combination with voluntary exercises (anti-resistant training) achieves the greatest improvements in muscle strengths and, therefore, in the recovery of the muscles required for swallowing.

With regard to the studies in this review, both stimulation types seem to be effective in the therapy of dysphagia. Thirteen out of 15 studies which reported to stimulate above motor threshold have found (restricted) beneficial outcomes in relation with NMES [4], [5], [6], [7], [8], [9], [10], [11], [12], [14], [15], [17], [19], and all of the four studies which stimulated at sensory threshold also reported positive outcomes in relation with NMES [12], [13], [18], [20].

## Conclusion

The purpose of this systematic review was to evaluate the latest studies regarding a potential effectiveness of NMES as a treatment for oropharyngeal dysphagia considering different aspects. It could generally be concluded that there is a considerable amount of level 2 studies which suggest that NMES is an effective treatment option when combined with TDT for patients with dysphagia after stroke and patients with Parkinson’s disease or with different kinds of brain injuries leading to dysphagia.

Up to date, not a single study has investigated or better defined the most effective NMES and therapy parameters, even though it is hypothesized that adequate protocols represent a key factor in the effectiveness of NMES therapy. A clear therapy suggestion concerning therapy parameters or therapy frequency can therefore not be derived from this systematic review.

Further research is necessary in order to clarify which stimulation protocols, parameters and therapy settings are most beneficial for certain patient groups and degrees of impairment.

## Limitations

In this systematic review, data pooling and statistical analysis could not be carried out due to the inhomogeneity of study protocols.

## Notes

### Authorship

Simone Miller and Katharina Peters have shared first authorship.

### Funding

As part of a third party funded project, this work was funded by the AiF Project GmbH of the BMWi (Germany’s Federal Ministry for Economic Affairs and Energy), KK5093801TS0.

### Competing interests

The authors work on a project concerning electrical stimulation and dysphagia (KK5093801TS0), which is funded by the Federal Ministry for Economic Affairs and Energy. This project is based on a cooperation with Physiomed Elektromedizin.

## Supplementary Material

Summary of the studies identified and matching the criteria to be included in this review

## Figures and Tables

**Table 1:**
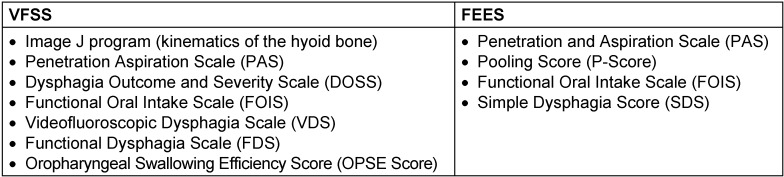
Overview of scales used with videofluoroscopy (VFSS) and fiberoptic endoscopic evaluation of swallowing (FEES)
