# Supplementary material for: Review of the effectiveness of neuromuscular electrical stimulation in the treatment of dysphagia – an update
Source: Ger Med Sci. 2022 Jun 14;20:Doc08. doi: 10.3205/000310 (PMC9284430; doi:10.3205/000310)
Supplement: Summary of the studies identified and matching the criteria to be included in this review [file GMS-20-08-s-001.pdf]

Attachment 1

Summary of the studies identified and matching the criteria to be included in this review

No.	Authors, year, title, type of study	Participants and comparisons	ES device and application period	Electrode placement and NMES parameters	Action during stimulation	Swallowing outcome measure	Results	Level of Evidence
1	**Konecny et al., 2018**<br>Electrical stimulation of hyoid muscles in post-stroke dysphagia.<br>randomized controlled trial	Patients with dysphagia following stroke (acute phase); experimental group (n=54): NMES + TDT; control group (n=54): TDT	not given<br>5 days a week, 20 mins per session, 4 weeks	suprahyoid region<br>TENS: 60 Hz<br>intensity: above motor threshold	no	VFSS: OTT, PTT	+<br>Significant changes in OTT and PTT after four weeks of rehabilitation in both, the study and the control group. The calculated differences were also significant with a greater benefit for the study group.	2
2	**Langmore et al., 2015 (2016 paper version)**<br>Efficacy of electrical stimulation and exercise for dysphagia in patients with head and neck cancer: A randomized clinical trial.<br>double-blinded, randomized controlled trial	Patients with dysphagia following head and neck cancer; experimental group (n=116/until Follow up 91): NMES + swallowing exercises; control group (n=54/until follow up: 36): sham NMES + swallowing exercises	BMR NeuroTech 2000 (Galway, Republic of Ireland)<br>6 days a week, 2 times per day, 20 mins per session (= 40 mins per day), 12 weeks	suprahyoid region<br>70 Hz, pulse width 300 msec (contraction: 4s, relaxation: 15 s, ramp up: 2s ramp down: 0s)<br>intensity: above motor threshold (comfortable contraction)	swallowing maneuvers	VFSS: evaluation of hyoid excursion, PAS Score, OPSE Score; diet (PSS); quality of life (HNCI)	–<br>The active NMES group had significantly worse PAS scales than the sham group. OPSE scores and hyoid excursion did not change significantly. Both groups reported significant better diet (PSS) and quality of life (HNCI).	2

**3**	**Simonelli et al., 2019**<br>A stimulus for eating. The use of neuromuscular transcutaneous electrical stimulation in patients affected by severe dysphagia after subacute stroke: A pilot randomized controlled trial.<br>pilot, single-blinded, randomized controlled trial	Patients with dysphagia following stroke (subacute phase); experimental group (n=17): NMES + TDT; control group (n=16): TDT	VitalStim: Intelect vitalstim<br>5 days a week, 30 mins per session, 8 weeks	infrahyoid region<br>80 Hz, pulse width 300 msec<br>intensity: ranged from 7.8 to 12.5 mA	TDT	primary outcome:FOIS; FEES: PAS, P-Score, presence of oropharyngeal secretion; secondary outcome: type of diet, need for postural compensations, duration of the dysphagia training	**(+)**<br>Functional improvement was observed in both groups but experimental group showed a significant higher improvement for primary outcome with exception of the P-Score and for secondary outcome.	2
**4**	**Park et al., 2018**<br>Effects of neuromuscular electrical stimulation in patients with Parkinson's disease and dysphagia: A randomized, single-blind, placebo controlled trial.<br>a single-blind, randomized, placebo controlled trial	Patients with dysphagia following Parkinson's disease; experimental group (n=9): NMES + effortful swallow + TDT; control group (n=9): sham NMES + effortful swallow + TDT	VitalStim<br>5 days a week, 30 min per session, 4 weeks	infrahyoid region<br>80 Hz, 2 channels of bipolar electric stimulation at fixed 80 Hz pulse rate and fixed pulse duration of 700 mys<br>intensity: above motor threshold (strong muscle contraction)	swallowing maneuver: effortful swallow	VFSS: image (evaluation of hyoid movement), VDS: evaluation of oral phase and pharyngeal phase, PAS	**(+)**<br>NMES increased hyoid bone movement (horizontal + vertical) and reduces aspiration (PAS) in the experimental group compared to control group. There were no significant differences in oral or pharyngeal phase of VDS.	2

**5**	**Park et al., 2016**	Patients with dysphagia following stroke (subacute phase); experimental group (n=31>25): NMES + effortful swallow +TDT; control group (n=30>25): sham NMES + effortful swallow +TDT	VitalStim (Chattanooga Group, Hixson, TN)	infrahyoid region	swallowing maneuver: effortful swallow	VFSS: image (evaluation of hyoid movement), VDS (evaluation of oral and pharyngeal stage), PAS	**+**	2
	Effects of neuromuscular electrical stimulation combined with effortful swallowing on post-stroke oropharyngeal dysphagia: a randomised controlled trial.		5 days a week, 30 min per session, 6 weeks	80 Hz, fixed biphasic pulse duration of 700 ls.			NMES increased hyoid bone movement (horizontal + vertical), reduces aspiration (PAS) and improves oral and pharyngeal phase of the swallowing function (VDS) in the experimental group. The control group showed significant differences in the total score and the oral phase (VDS). Significant differences between groups in the total score and the pharyngeal phase (VDS) and on the PAS.	
	single-blind, randomized, controlled trial; comparative study			intensity: above motor threshold (strong muscle contraction), sham: 1.0 mA				

6	**Meng et al., 2018**<br>The effect of surface neuromuscular electrical stimulation on patients with post-stroke dysphagia.<br>a pilot, randomized, controlled trial	Patients with dysphagia following stroke (acute and subacute phase); three study arms (n=10 each): two experimental groups (NMES+TDT): 1.TGA: NMES applied to suprahyoid and infrahyoid region, 2. TGB: NMES applied to suprahyoid region; one control group: TDT	VitalStim hand held device (DJO Global, Inc. Vista, CA, USA)<br>5 days per week, 30 min per session, 10 treatments	other: vertical + horizontal; Group A (suprahyoid and infrahyoid regions), GROUP B (only suprahyoid region)<br>80 Hz<br>intensity: above motor threshold (comfortable contraction); wave amplitude 0-25 mA.	swallow during sNMES	VFSS: evaluation of hyoid movement, DOSS, WST, RSST	**+**<br>Swallowing function (WST, RSST, DOSS) was significantly improved in all three groups but there was a significant higher improvement between the study groups with no inter-group differences of TGA and TGB. However, NMES on suprahyoid region could further improve the moving distance of hyoid bone anteriorly.	2
7	**Sproson et al., 2018**<br>Combined electrical stimulation and exercise for swallow rehabilitation post-stroke: a pilot randomized control trial.<br>pilot study, randomized, controlled	Patients with dysphagia following stroke (subacute phase); experimental group: Ampcare ESP (n=15); control group (n=15): TDT	Ampcare's ESP<br>5 days a week, 30 min per session, 4 weeks	suprahyoid region<br>30 Hz	TDT: swallow-strengthening exercises	VFSS: PAS, SWAL-QoL, FOIS	+<br>75% (9/12) of the experimental group compared with 57% (8/14) of the control group improved. Comparative benefit of 1.5 on the FOIS, and on the PAS: 1.35 for diet and 0.3 for fluids. The intervention group also reported much better outcome satisfaction.	2

8	**Guillen-Sola et al., 2017**	Patients with dysphagia following stroke (subacute phase); two experimental groups: group III (n=20): TDT+ sham IEMT+ NMES, group II (n=21): TDT + ITEM; control group: group I (n=21): TDT	VitalStim, Chattanooga Group, Hixson, TN, USA	suprahyoid region	swallow during sNMES	VFSS: FOIS Scale, PAS and DOSS; VVST, signs of security and efficacy of swallowing	**(0/+)**	2
	Respiratory muscle strength training and neuromuscular electrical stimulation in subacute dysphagic stroke patients: a randomized controlled trial.		5 days a week, 40 min per session, 3 weeks	80 Hz			At the end of intervention: Swallowing security signs were improved in Groups II and III; At 3 month follow up: No differences in PAS Scale or respiratory complication were detected.	
	a randomized controlled trial			intensity: above motor threshold (perceived muscle contraction)				
9	**Zeng et al., 2018**	Patients with dysphagia following stroke (acute phase); experimental group (n=59): NMES + TDT; control group (n= 53): TDT	YS1002T Glossopharyngeal Nerve and muscle electrical stimulator (Changzhou Yasi Medical Instruments Co., LTD)	other (both regions): vertical arrangement	no	Kubota water drinking test, HAMA Scale, HAMD Scale	**+**	2
	Efficacy of neuromuscular electrical stimulation in improving the negative psychological state in patients with cerebral infarction and dysphagia.		12 days, 20 mins per session, 3 days break, another 12 days, 20 mins per session	Frequency: no information; pulse width of 800 ms			The rate of swallowing improvement: 88.1% in the experimental group, 69.8% in the control group; HAMA Scale and HAMD Scale: significant improved in the experimental group compared to the control group.	
	a randomized controlled trial			intensity of 28 mA				

10	**Maeda et al., 2017**<br>Interferential current sensory stimulation improves airway defence and oral nutrition intake in patients with dysphagia: a double-blind randomized controlled trial.<br>a double-blind randomized controlled trial	Patients with dysphagia following different diseases: experimental group (n=22): NMES + TDT; control group (n=21): sham NMES + TDT	Gentle Stim®; J Craft, Osaka, Japan<br>5 days a week, 2 times per day, 15 mins per session (40 mins per day), 2 weeks	other<br>50 Hz, two pairs of electrodes of different frequencies (2,000 and 2,050 Hz) across the neck generating a 50-beat interferential current<br>intensity: sensory stimulation set at 3.0 mA; sham stimulation set at 0.1 mA	no	cough latency times against a 1% citric acid mist, FOIS, oral nutritional intake (kcal/day)	**+**<br>Experimental group: positive changes in cough latency time at two weeks (−14.1±14.0 s vs −5.2±14.2 s, p=0.047) and oral nutrition intake at three weeks (437±575 vs. 138±315 kcal/day, p=0.042); Between group differences were not significant concerning all outcome parameter (cough latency, cough frequency, FOIS, nutritional oral intake (kcal per day).	2
11	**Oh et al., 2020**<br>The effect of neuromuscular electrical stimulation with different electrode positions on swallowing in stroke patients with oropharyngeal dysphagia: A randomized trial.<br>a randomized trial, comparative study	Patients with dysphagia after stroke (n=38) (sub-acute phase); two experimental groups: 1. SMG + TDT (SMG = suprahyoid muscle group) (n=18), 2. IMG + TDT (IMG = infrahyoid muscle group) (n=20)	VitalStim (Chattanooga Group, Hixson, TN)<br>5 days a week, 30 min per session, 4 weeks	suprahyoid vs. infrahyoid region<br>80 Hz, biphasic pulse duration 700 msec,<br>intensity: above motor threshold (grabbing sensation), 9.0 - 14.0 mA	swallowing maneuver: effortful swallow	VFSS: VDS, PAS, FOIS	**+**<br>Both groups showed significant improvements in oropharyngeal function and level of functional oral intake (no significant difference between groups). SMG showed more reduced PA compared to IMG.	2

12	**Zhang et al., 2016**<br>Effectiveness of Neuromuscular Electrical Stimulation on Patients with Dysphagia and with Medullary Infarction.<br>a randomized trial, comparative study	Patients with dysphagia with medullary infarction (acute phase); two experimental groups: 1. motor NMES + TDT (n=27), 2. sensory NMES + TDT (n=28); one control group: TDT (n=27)	vocaSTIM-Master<br>5 days a week, 2 times a day, 20 min per session (= 40 mins per day), 4 weeks	other: sensory: cathode was placed on the submental region, and the anode was placed on the occipital skin. motor: cathode and anode were placed in parallel on the skin of the anterior belly of the digastric muscle in the submental region above the hyoid bone<br>T/R exponential current: sensory: frequency of 25 Hz, pulse width of 1 s; motor: frequency of 120 Hz, pulse width of 100 ms<br>intensity: sensory: input level expected to lead to swallowing; motor: motor threshold	no	WST, standardized swallowing assessment, FOIS, SWAL-QOL	+<br>The sensory approach group showed significantly greater improvement than the other two groups. The motor approach group showed greater improvement than the TDT group.	2
13	**Umay et al., 2017**<br>The effect of sensory level electrical stimulation of the masseter muscle in early stroke patients with dysphagia: A randomized controlled study.<br>a randomized controlled trial	Patients with dysphagia following stroke (acute phase); experimental group (n=58): NMES + TDT; control group (n=40): sham NMES + TDT	Intelect Advanced-Chattonooga, UK<br>5 days a week, 60 min per session, 4 weeks	other: bilateral masseter muscles<br>intermittent galvanic stimulation; amplitude of the current: 4-6 mA<br>intensity: sensory threshold	no	BDS, NEDS, TDS, MASA; evaluation of the dysphagia level by FEES; FIM	**+**<br>There were significant improvement in dysphagia severity scores (evaluated by BDS, NEDS, TDS, MASA and FEES) and in cognitive and total functionality levels (FIM) in the stimulation group. In the sham group, there were no significant changes in the evaluation parameters.	2

**14**	**Carnaby et al., 2020**<br>Exercise-based swallowing intervention (McNeill Dysphagia Therapy) with adjunctive NMES to treat dysphagia post-stroke: A double-blind placebo-controlled trial.<br>a double-blind randomized placebo-controlled trial	Patients with dysphagia following stroke (subacute phase); two experimental groups: 1. (n=18): NMES + MDTP (MCNEILL Therapy), 2. (n=18): sham NMES + MDTP; one control group (n=17): TDT	VitalStim® system.<br>7 days a week, 60 mins per session, 3 weeks	other (both regions): vertical arrangement<br>not specified<br>intensity: motor threshold	MDTP combined with NMES	MASA score; FOIS; MBS outcomes (dysphagia and aspiration; yes/no, %); patient self-perception of swallowing ability; body weight, time to recover pre-stroke diet, occurrence of dysphagia–related health complications	**(+)**<br>Post treatment dysphagia severity and treatment response were significantly different between groups ($p \leq 0.0001$). MDTP demonstrated greater positive change than either NMES or UC arms (MASA Score, MBS outcome), including increase in oral intake ($\chi2=5$, $p \leq 0.022$) and improved functional outcome by 3-months post stroke (RR = 1.72, 1.04–2.84).	2

15	**Poorjavad et al., 2019**<br>Effects of the head lift exercise and neuromuscular electrical stimulation on swallowing muscles activity in healthy older adults: a randomized pilot study.<br>a randomized pilot study	Older adults; Two experimental groups: 1. (n=11): NMES, 2. (n=12): HLE ; no control group	dual-channel electrotherapy device (FarMed, Tehran, Iran)<br>5 days a week, three times a day, 15 mins per session with 5-minute rest intervals (= 45 mins per day), 2 weeks	suprahyoid region<br>frequency of 100 Hz with a stimulus duration of 1 to 7 s and 8-s resting intervals (aim: increasing the tolerance level)<br>intensity: maximal tolerable	no	pre- and post-therapy surface electromyography (sEMG) during water swallowing	–<br>For the HLE group, duration of suprahyoid muscles activity was significantly reduced at post-intervention compared to pre-intervention (sEMG; p=0.036). After treatments, duration and latency between onset and peak amplitude of suprahyoid muscles activity was significantly shorter in the HLE group compared to the NMES group (sEMG: p=0.007 (duration), p=0.003 (latency))	2
16	**Terre and Mearin, 2015**<br>A randomized controlled study of neuromuscular electrical stimulation in oropharyngeal dysphagia secondary to acquired brain injury.<br>a pilot randomized controlled trial	Patients with dysphagia after acquired brain injury (Stroke + STBI) (sub-acute phase); experimental group (n=10): NMES + TDT; control group (n=10): sham NMES + TDT	VitalStim<br>5 days a week, 60 mins per session, 2 weeks	both regions: 2 sets of electrodes<br>80 Hz, pulse duration 300 µs<br>intensity: above motor threshold (maximal tolerable)	TDT	VFSS: OTT, PTT etc., FOIS; oesophageal manometry; patient satisfaction (Likert scale)	–<br>For the HLE group, duration of suprahyoid muscles activity was significantly reduced at post-intervention compared to pre-intervention (sEMG; p=0.036). After treatments, duration and latency between onset and peak amplitude of suprahyoid muscles activity was significantly shorter in the HLE group compared to the NMES group (sEMG: p=0.007 (duration), p=0.003 (latency)).	2

**17**	**Huang et al., 2014**<br>Functional outcome in acute stroke patients with oropharyngeal Dysphagia after swallowing therapy.<br>a pilot randomized controlled trial	Patients with dysphagia following stroke (acute phase); two experimental groups: 1. (n=8): NMES, 2. (n=10): NMES + TDT; one control group (n=11): TDT	VitalStim<br>3 days a week, 60 min per session, 10 sessions	infrahyoid: vertical arrangement<br>80 Hz, pulse width of 700 µs<br>intensity: above motor threshold (tolerance level)	no	clinical swallowing assessments; VFSS: functional dysphagia scale (FDS), FOIS, PAS (baseline + after treatment)	(+)<br>TDT and TDT+NMES both had significant swallowing improvement after therapy. In acute stroke patients with dysphagia, TDT+NMES is the most effective swallowing therapy in taking solid diets and thick liquids.	2

18	**Ortega, et al., 2016**<br>A Comparative Study Between Two Sensory Stimulation Strategies After Two Weeks Treatment on Older Patients with Oropharyngeal Dysphagia.<br>a randomized trial	Elderly patients with dysphagia; two experimental groups: 1. Group A (n=19): transient receptor potential vanilloid 1 (TRPV1) agonist, 2. Group B (n=19): transcutaneous sensory electrical stimulation (TSES); no control group	Intelect VitalStim device (Chattanooga Group, Hixson, TN, USA)<br>5 days a week, 60 mins per session, 2 weeks	infrahyoid region: thyrohyoid position<br>80 Hz, biphasic pulses, 300 µs (Abstract) or 700 µs (method)<br>intensity: sensory threshold (75% of motor threshold)	no	clinical symptoms: EAT - 10 score; VFFS: signs of OD: prevalence of ISS (PAS Score) and IES (OR and PR); OSR parameters; response to the treatment (percent of responders/non responders)	**(+)**<br>Post treatment benefits regarding clinical symptoms: TRPV1 agonists induced a significant reduction (EAT 10 score). VFS signs of OD were significantly and similarly reduced in both groups. No differences were found regarding OSR parameters and hyoid kinematics. Response to treatment: There were 68.42% responders in Group A (TRPV1) and 42.11% in Group B (TSES). Group A responders showed an improvement in PAS (PAS, 5.23 ± 2.04 to 3 ± 1.47; P = 0.002), and the same was true for those of Group B (4.63 ± 1.41 to 2.13 ± 0.64; P = 0.007).	2

NMES: neuromuscular electrical stimulation, TDT: traditional dysphagia therapy, VFSS: Videofluoroscopic Swallowing Studies, OTT: oral transit time, PTT: pharyngeal transit time, PAS: Penetration and Aspiration Scale, OPSE: oropharyngeal swallowing efficiency, PSS: Performance Status Scale, HNCI: Head and Neck Cancer Inventory, FOIS: Functional Oral Intake Scale, FEES: Fiberoptic Endoscopic Examination of Swallowing, P-Score: Pooling Score, VDS: Videofluroscopic Dysphagia Scale, DOSS: Dysphagia Outcome and Severity Scale, WST: Water Swallow Test, RSST: Repetitive Saliva Swallowing Test, VVST: Volume Viscosity Swallow Test, ESP: Effective Swallowing Protocol, VVST: Volume Viscosity Swallow Test, HAMA Scale: Hamilton anxiety scale, HAMD Scale: Hamilton depression scale, NEDS: Neurological Examination Dysphagia Score, BDS: Bedside Dysphagia Score, NEDS: Neurological Examination Dysphagia Score, TDS: Total Dysphagia Score, MASA: Mann Assessment of Swallowing Ability Test, FIM: Functional Independence Measure, MDTP: MCNEILL Therapy, HLE: head lift exercise, MBS: modified barium swallow, PR: pharyngeal residues, OR: oral residues, ISS: impaired safety of swallow, IES: impaired efficacy of swallow, OSR: oropharyngeal swallow response
